# Socioeconomic Gender Variables Impact the Association between Hypertension and Chronic Health Issues: Cross-Sectional Study

**DOI:** 10.3390/jpm14080890

**Published:** 2024-08-22

**Authors:** Simon David Lindner, Teresa Gisinger, Peter Klimek, Alexandra Kautzky-Willer

**Affiliations:** 1 Institute of the Science of Complex Systems, CeMSIIS, Medical University of Vienna, Waehringer Guertel 18-20, 1090 Vienna, Austria; simon.lindner@meduniwien.ac.at (S.D.L.); peter.klimek@meduniwien.ac.at (P.K.); 2 Complexity Science Hub Vienna, Josefstädter Straße 39, 1080 Vienna, Austria; 3 Gender Medicine Unit, Department of Internal Medicine III, Division of Endocrinology and Metabolism, Medical University of Vienna, 1090 Vienna, Austria; teresa.gisinger@meduniwien.ac.at; 4 Supply Chain Intelligence Institute Austria, Josefstädter Straße 39, 1080 Vienna, Austria; 5 Department of Clinical Neuroscience, Division of Insurance Medicine, Karolinska Institute, 171 77 Stockholm, Sweden

**Keywords:** gender medicine, hypertension, comorbidities, cardiovascular risk

## Abstract

Our aim is to investigate if sex and gender influence the association of hypertension and their comorbidities. We investigated how gender differences in five socioeconomic factors impact the relation between hypertension and ten comorbidities including diabetes mellitus, renal disease, and chronic pulmonary disease in European countries grouped by their gender inequality index using representative survey data from the European Health Interview Survey. Using logistic regressions, we compute the ratio of odds ratios in females versus males. Therefore, an ORR > 1 is associated with a higher odds ratio for females than for males while an ORR < 1 means the opposite. To account for multiple hypothesis testing, we applied the Bonferroni correction. Hypertension in both sexes was associated with lower educational level, being unemployed, and lower income. In males, being divorced/widowed (OR1.12, *p* < 0.001) had an association to hypertension, whereas in females, being common-law/married (OR1.30, *p* < 0.001) and being divorced/widowed (OR1.17, *p* < 0.001) was associated with a higher risk for hypertension. Moreover, in hypertension, females who worked had an association with myocardial infarction (OR1.39, *p* < 0.001) and having post-secondary education had an association with arthrosis (OR 1.35, *p* < 0.001) compared to males. Our findings show that gender variables influence the association of hypertension and comorbidities, especially in females. These results can be used to inform targeted prevention measures taking gender-specific contextual factors into account.

## 1. Introduction

Worldwide hypertension is the leading preventable risk factor for all-cause mortality and cardiovascular diseases [1,2]. In 2010, reports counted 1.39 billion people with hypertension, which accounts for 31.1% of the global adult population [3]. In recent years, the prevalence of hypertension has been steadily increasing [3]. Some of the assumed reasons are the ageing of the population and increasing exposure to lifestyle risk factors including unhealthy diet, high sodium and low potassium intake, and lack of physical activity [3]. Moreover, hypertension is an important risk factor for diseases as heart failure and other cardiovascular diseases [4,5]. Further, hypertension is often related to unhealthy lifestyle behaviors, such as smoking, diabetes mellitus, dyslipidemia, overweight, physical inactivity, and unhealthy diet [5]. Moreover, it has already been reported that the risk for hypertension varies between low-, middle-, and high-income countries [6]. This could be caused by differences in health-promoting environments such as salt-reduction policies, sugar and alcohol tax, or low-cost single-pill combination therapy to improve adherence [6].

It is already known that there are sex differences concerning the prevalence of hypertension [7]. Previous data reported that males have a higher prevalence of hypertension than females among adults aged 18–39 years and 40–59 years [7]. Nevertheless, in the age group older than 60 years, a lower prevalence of hypertension in males compared to females can be found [7]. Socioeconomic variables, such as educational level, are known to influence health but also are influenced by sociocultural gender [8]. Past literature provided insights suggesting that education may be considered a good predictor of global cardiovascular risk in individuals with hypertension [9]. Considering adherence to antihypertensive drug treatment, a higher proportion of days covered with antihypertensive treatment was reported among men and couples living together [10].

Nevertheless, little is known about the influence biological sex and socio-cultural gender have on hypertension-related comorbidities. Therefore, we investigated if gender-related variables impact the relation of hypertension and comorbidities and if it differs between different European countries, which vary in income level and healthcare systems, and between males and females.

## 2. Materials and Methods

### 2.1. Data

For the analysis, the European Health Interview Survey (E-HIS-second wave, 2013–2015, N = 316,333, females: 51.3%) was used. The European health information survey (EHIS) runs every 5 years. The first wave was conducted between 2006 and 2009, and a second wave was conducted between 2013 and 15 in all European Union member states at the time as well as Iceland and Norway. The countries included in the second wave were specifically as follows: 2013: Belgium and the United Kingdom; 2014: Bulgaria, Czechia, Estonia, Greece, Spain, France, Croatia, Italy, Cyprus, Latvia, Lithuania, Luxembourg, Hungary, Malta, Netherlands, Austria, Poland, Portugal, Romania, Slovenia, Slovakia, Finland, and Sweden; 2015: Denmark, Germany, Ireland, Iceland, and Norway. The study was conducted according to the guidelines of the Declaration of Helsinki and approved by the Committee of the Medical University of Vienna (protocol code 1859/2019 and date of approval 3 September 2019).

### 2.2. Statistical Analysis

The dataset was categorized based on the Gender Inequality Index (GII) from 2015 into three approximately equally sized groups:

1. Low GII countries: Denmark, Sweden, Netherlands, Norway, Belgium, Finland, Slovenia, Iceland, Germany, Luxembourg, and Spain.
2. Medium GII countries: France, Italy, Austria, Portugal, Cyprus, Greece, Ireland, and Lithuania.
3. High GII countries: Czechia, Poland, Estonia, United Kingdom, Croatia, Slovakia, Malta, Latvia, Bulgaria, Hungary, and Romania.

Beyond these groupings, both the full dataset and the individual GII categories were further stratified by sex. This division resulted in eight distinct strata: one for the entire dataset divided by sex, and six based on the GII-sex combination.

Multivariate regression analysis was undertaken on the entire dataset (stratified by sex) and within each of the six GII-sex strata. Each regression model had one of the following medical conditions as the outcome variable: asthma, chronic pulmonary disease, myocardial infarction, cardiovascular disease, angina pectoris, hypertension, stroke, arthrosis, or diabetes mellitus (see Supplementary Table S1).

The independent variables included in each model were age, household size, income, working status over the last 12 months, marital status, and education, all of which are gender-related socioeconomic factors. For each of these variables, interactions with hypertension were tested. Specifically, we analyzed a regression model across all three GII categories and a general cohort, stratified by sex, for 10 different health conditions, resulting in a total of 80 models. This approach allowed us to systematically evaluate the interactions of five gender-related variables with 10 distinct health conditions in relation to hypertension. To account for multiple hypothesis testing, we applied the Bonferroni correction. If an interaction was deemed significant (with a family-wise error rate of less than 0.05) after post-Bonferroni correction for either males or females, we calculated the odds ratio (ORR = OR_F/OR_M). Subsequently, the significance of the ORR was assessed using the Welch’s *t*-test.

## 3. Results

In Table 1, we report the frequency of the socioeconomic and disease characteristics of the general cohort and the male and female cohorts. In Table 2, the association of gender-related variables with hypertension is reported in the general, female, and male cohorts. It could be noted that medium and high GII is associated with hypertension (OR 1.25, *p* < 0.001, OR 1.55, *p* < 0.001). Post-secondary education had a negative association with hypertension risk (OR 0.84, *p* < 0.001). On the other hand, being common-law/married (OR 1.21, *p* < 0.001), divorced/widowed (OR 1.13, *p* < 0.001), or having a medium (OR 1.13, *p* < 0.001) or low income (OR 1.17, *p* < 0.001) had a positive association with hypertension, whereas lower hypertension risk was associated with being employed (OR 0.76, *p* < 0.001) or having a household size of 3 or more (OR 0.86, *p* < 0.001). The same analyses were carried out for the female and male cohorts, where similar trends can be reported. The only sex difference was that only in the female cohort was being married associated with hypertension (OR 1.3, *p* < 0.001). Furthermore, a stronger association of high GII with hypertension could be found in females (OR 1.74, *p* < 0.001) compared to males (OR 1.34, *p* < 0.001).

In Table 3, the significant interactions between the socioeconomic variable and hypertension with health variables as outcome are reported. Chronic pulmonary disease was associated with hypertension in people with working status in the general cohort (OR 1.60, *p* < 0.001). Moreover, myocardial infarction was associated with hypertension in people who worked in the last 12 months (OR 1.39, *p* < 0.001), as well those with a as a household size of 3 or more (OR 1.40, *p* < 0.001). In depression, an association of hypertension with individuals with a household size of 3 or more (OR 1.57, *p* < 0.001), being divorced/widowed (OR 0.75, *p* < 0.001), and common-law/married (OR 0.73, *p* < 0.001) could be found. Concerning individuals with hypertension and who were common-law/married, an association with a lower risk of renal disease could be found (OR 0.65, *p* < 0.001) compared to being single. Furthermore, arthrosis was positively associated with hypertension in individuals with post-secondary education (OR 1.17, *p* < 0.001), a household size of 3 or more (OR 1.46, *p* < 0.001), and who were employed (OR 1.33, *p* < 0.001). A negative association of arthrosis was seen with being divorced/widowed (OR 0.74, *p* < 0.001) or common-law/married (OR 0.68, *p* < 0.001) in individuals with hypertension.

Concerning the GII-dependent analyses, we saw an association of myocardial infarction with hypertension in individuals who worked in the past 12 months (OR 1.64, *p* < 0.001) in the GII1 cohort. Moreover, in the GII1 cohort, individuals with hypertension and who were widowed or divorced had a negative association with chronic pulmonary disease (OR 0.32, *p* < 0.001) and arthrosis (OR 0.57, *p* < 0.001). In the GII2 cohort, depression had an association with hypertension and being widowed/divorced (OR 0.78, *p* = 0.004). Lastly, in the GII3 cohort, myocardial infarction was associated with hypertension in individuals who worked (OR = 1.51, *p* < 0.001). Moreover, in the GII3 cohort, depression was associated with hypertension in individuals with a household size of 3 or more (OR = 1.85, *p* < 0.001) and who are common-law/married (OR = 0.69, *p* < 0.001). In the GII3 cohort, chronic pulmonary disease was associated with hypertension in individuals with a high household income (OR = 1.35, *p* < 0.001) and who were employed (OR = 1.93, *p* < 0.001). In addition, in the GII3 cohort, myocardial infarction was associated with hypertension in individuals who are employed (OR = 1.51, *p* < 0.001).

**Figure 1:**
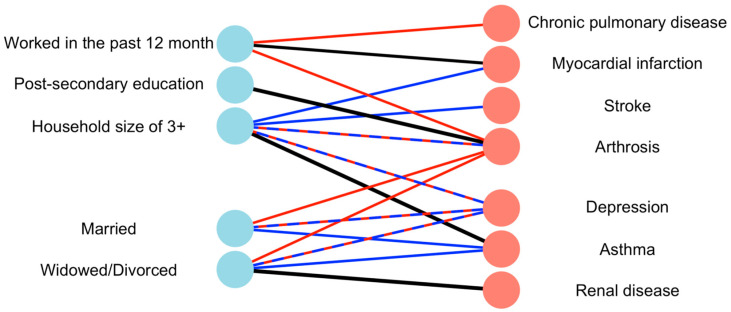
Summary of every interaction with a significant OR for females (red) and males (blue) and both (dashed red and blue line). For significant gender differences (ORR), the link is black (*p* < 0.05). Notes: Abbreviations: GII = Gender Inequality Index, EHIS= European Health Information Survey.

Lastly, we summarize all the interactions with significant difference between the male and female cohorts (Figure 1). Hypertension in females was associated with a stronger risk increase for arthrosis (ORR = 1.32, *p* < 0.001) in persons with post-secondary education. Moreover, hypertension had an association with asthma in females with a household size over 3 (ORR = 1.54, *p* < 0.001) and with renal disease in females who are divorced/widowed (ORR = 1.54, *p* < 0.001). After stratifying by GII, in countries in the GII category 2, hypertension was associated with a stronger risk increase for arthrosis in females living in a household of size 3 (ORR = 1.77, <0.001). In the GII category 3, hypertension was negatively associated with arthrosis in males living in a household size of 3 or more (ORR 0.59, *p* < 0.05).

## 4. Discussion

In summary, our study could show complex interactions between hypertension and gender-related variables; furthermore, hypertension is associated with other comorbidities and these associations are again influenced by gender-related variables. Moreover, the interaction of gender-related variables with the influence of hypertension on comorbidities was different between the two biological sexes. In general, gender-related variables such as household size, marital status, and educational level had more impact in the female population.

Concerning hypertension, a previous work already reported that lower socioeconomic status is associated with higher prevalence of this disease [11]. In our study, we analyzed gender-related characteristics which are also related to socioeconomic status. Thus, we could report that a higher prevalence of hypertension was associated with medium and low income levels. Furthermore, being employed and post-secondary education were associated with lower prevalence of hypertension. In addition, our work could also provide knowledge to further gender-related variables including marital status and household size, which are nowadays often neglected in research. A higher prevalence of hypertension was associated with being married and divorced/widowed. In contrast, a lower prevalence of hypertension was associated with a household size of 3 or more in our study. Lastly, our work also investigated differences between females and males regarding hypertension risk. Our findings suggest that a higher prevalence of hypertension was associated with being married and living in countries with a higher GII only in females, not in males. One of the suggested causes leading to higher hypertension incidence in individuals with lower socioeconomic status is dietary habits [11]. In particular, salt intake influences hypertension epidemiology [12]. Past literature reported an association of the intake of snacks with fatty and salty content and a higher prevalence of hypertension, which was more likely seen in individuals with lower socioeconomic status [11]. Also, other unhealthy habits such as cigarette smoking, alcohol consumption, and less physical activity are associated with low socioeconomic status [13]. Furthermore, a higher awareness of predictive measures against hypertension is related to higher socioeconomic status [14]. Moreover, education level also plays a role in the risk of hypertension [11]. In both females and males, a higher risk of hypertension with lower educational level was reported [11]. A German and Czech study found that unemployment also increases the risk for hypertension [11]. These findings are in line with our previously mentioned results.

Hypertension is also related to various comorbidities including coronary artery disease, myocardial infarction, congestive heart failure, stroke, and chronic kidney disease [15,16]. Moreover, some studies suggest an association of hypertension and asthma as well as arthrosis [17,18,19]. Still, the awareness of hypertension complications within the population is low, and even in young individuals with hypertension, more than two hypertension-related end-organ damages can be found [20,21].

Nevertheless, the novelty of our study was to investigate the association of gender-related variables with the risk of comorbidities in individuals with hypertension. In our study, we were able to find that working status was correlated with numerous comorbidities in participants with hypertension. More specifically, a higher frequency of chronic pulmonary disease, myocardial infarction, and arthrosis was associated with being employed in individuals with hypertension. A reason for this could be the higher stress level which one experiences at work [22]. Moreover, taking care of children can also cause stress [23]. Past work reported that individuals with high stress from caregiving responsibilities have a higher percentage of saturated fat intake compared to individuals with lower stress [23]. Furthermore, an US study found that only women without stress were able to obtain an ideal diet based on serving sizes of whole grains, salt, fruits and vegetables, protein, and sugary drinks [24]. This could explain why females with hypertension had a higher association with renal disease compared to males when widowed/divorced. However, females in comparison to males mostly have a wider social network besides their family, and in the literature, marital status is often used as a measurement of social support [25,26]. Nevertheless, elevated stress levels could contribute to a higher incidence of comorbidities in widowed or divorced females with hypertension. These women often bear the dual responsibilities of caring for their children and working to ensure their financial stability. Furthermore, studies have shown that married females tend to shoulder a greater proportion of household and childcare responsibilities compared to their male counterparts. Therefore, married men experience less stress beside their workplace [25,26]. Life stress such as occupational stress or post-traumatic stress are associated with higher prevalence of hypertension and, in addition, cardiovascular diseases [27]. In our study, we could report that only females with hypertension experience a higher rate of myocardial infarction when they are employed compared to employed males, which could also be caused by the increased stress levels.

A specific disease which is known to be more prevalent in females is depression. Past literature already reported an association of hypertension and depression [28]. Moreover, the combination of depression, hypertension, and stroke was higher in females compared to males [29,30,31]. It is postulated that individuals with hypertension and depression have an over-acting sympathetic nervous system. Therefore, it could be claimed that stroke, depression, and hypertension share a common pathological pathway and, furthermore, have a higher risk to occur together [32]. In our study, we could show that a household size of 3 or more had a positive association with depression and hypertension in both sexes. Also, stroke had an association with having a household size of 3 or more in individuals with hypertension in our study. On the other hand, being divorced/widowed and being common-law/married had a negative association with depression in females and males with hypertension. Nevertheless, this effect was more profound in the female cohort, even though the difference is not significant. As mentioned before, being divorced/widowed might influence a person’s mental health as it is related to less social interaction and also more stress through housework and caregiving responsibilities. Still, being common-law/married is also associated with depression in individuals with hypertension in our study. Previous literature could already report higher rate of depression in married females, but not in males [33].

Lastly, we could investigate that more sex-specific differences in the influence of gender on hypertension comorbidities are seen in countries with higher gender inequality. More specifically, females were more likely to have an association of gender-related variables with hypertension comorbidities. This phenomenon could be caused by worse access to health care for females in countries with higher gender inequality or by the higher stress females experience in these countries as males do not feel a responsibility to help with household activities or childcare. Moreover, in countries where males are gatekeepers to the health care system, females report worse health outcome as they have issues accessing the health care system or feel uncomfortable seeking help [34].

Our findings should be interpreted considering some limitations. The first is that we used self-reported data. Indeed, the usage of self-reported data could lead to over- and underestimated frequency of diseases. Furthermore, no gender dimensions were asked in the health survey. Lastly, crucial variables such as sex hormone levels or other biochemical markers are missing in this database, which would be important to better understand the effect of sex and gender on hypertension outcome and increase the clinical significance.

## 5. Conclusions

The novelty of our study is that we investigated not only biological sex but also sociocultural gender in respect to hypertension comorbidities. Therefore, our work could provide knowledge on how to better support individuals with hypertension, especially females.

## Figures and Tables

**Table 1:** The dataset comprises a total of N = 316,333 entries, with 171,399 entries corresponding to females and 144,934 entries corresponding to males. Among these entries, a subgroup of N = 74,748 entries (42,286 females and 32,462 males) specifically pertain to a report on hypertension. Characteristics of study sample of individuals with hypertension stratified by sex.

	Female	Male	Overall
	N = 42,231	N = 32,424	N = 74,655
Household size of 1	33.30%	18.30%	26.80%
Household size of 2	41.70%	50.60%	45.60%
Household size of 3	24.90%	31.10%	27.60%
	N = 42,179	N = 32,361	N = 74,540
Single	7.90%	12.30%	9.80%
Married	41.30%	15.90%	30.30%
Widowed/Divorced	50.80%	71.70%	59.90%
	N = 42,061	N = 32,311	N = 74,372
Working in the last 12 months	21.60%	34.10%	27%
	N = 39,662	N = 30,649	N = 70,311
Income Level: 1st third	29.20%	40.20%	34%
2nd third	22%	21.90%	22%
3rd third	48.80%	37.90%	44.10%
	N = 41,979	N = 32,263	N = 74,242
Secondary education	82.30%	76%	79.60%
Post-secondary education	17.70%	24%	20.40%
Asthma	N = 41,524	N = 31,793	N = 73,317
	8.40%	8.30%	8.30%
Chronic pulmonary disease	N = 41,458	N = 31,765	N = 73,223
	3.60%	6.80%	5%
Myocardial infarction	N = 41,473	N = 31,768	N = 73,241
	12.40%	12.30%	12.40%
Stroke	N = 41,595	N = 31,803	N = 73,398
	38.30%	22.80%	31.60%
Arthrosis	N = 41,681	N = 31,959	N = 73,640
	18.30%	19.60%	18.90%
Diabetes mellitus	N = 41,675	N = 31,879	N = 73,554
	18.60%	13.30%	16.30%
Cirrhosis of the liver	N = 41,570	N = 31,798	N = 73,368
	0.80%	1.10%	0.90%
Urinary incontinence	N = 41,697	N = 31,860	N = 73,557
	14.80%	9.30%	12.40%
Renal disease	N = 41,589	N = 31,822	N = 73,411
	7.90%	6.60%	7.30%
Depression	N = 41,604	N = 31,844	N = 73,448
	14.40%	9%	12.10%

**Table 2:** Shows the results of the regression conducted with hypertension as the outcome variable and GII category and the socio-economic gender variables as explanatory variables adjusted for age.

	OR (95% CI)	OR (95% CI) Female	OR (95% CI) Male
Medium GII category	1.25 (1.21, 1.29) **	1.26 (1.21, 1.31) **	1.24 (1.19, 1.29) **
High GII category	1.55 (1.49, 1.61) **	1.74 (1.66, 1.83) **	1.34 (1.27, 1.41) **
Post-secondary education	0.84 (0.82, 0.86) **	0.78 (0.75, 0.8) **	0.92 (0.89, 0.95) **
Household size of 2	0.97 (0.94, 1)	0.97 (0.93, 1.02)	1.01 (0.96, 1.06)
Household size of 3+	0.86 (0.83, 0.9) **	0.87 (0.83, 0.91) **	0.88 (0.83, 0.93) **
Medium Income Level	1.13 (1.1, 1.16) **	1.17 (1.13, 1.22) **	1.09 (1.05, 1.13) **
Low Income Level	1.17 (1.14, 1.2) **	1.27 (1.23, 1.31) **	1.06 (1.03, 1.1) **
Married	1.21 (1.16, 1.25) **	1.3 (1.23, 1.37) **	1.01 (0.96, 1.07)
Widowed/Divorced	1.13 (1.09, 1.17) **	1.17 (1.1, 1.23) **	1.12 (1.06, 1.18) **
Worked in the past 12 m	0.76 (0.74, 0.78) **	0.75 (0.72, 0.77) **	0.76 (0.73, 0.79) **

** *p* < 0.001.

**Table 3:** Summary of the significant interactions between the socioeconomic variable and hypertension with health variables as outcome. We found 47 significant interactions, of which 29 hold in the full dataset, 4 hold for low GII countries, 9 for medium GII countries and 5 for high GII countries. The most common health issue we found is diabetes mellitus with 13 occurrences, followed by depression with 9 occurrences.

Disease	Gender Variable	OR (95% CI)	OR (95% CI) Female	OR (95% CI) Male	ORR
Full Dataset and adjusted for GII					
Asthma	Household size of 3+	1.41 (1.23, 1.61) **	1.67 (1.4, 1.98) **	1.08 (0.87, 1.35)	1.54 **
Asthma	Married	0.65 (0.56, 0.74) **	0.61 (0.51, 0.74) **	0.76 (0.61, 0.95)	0.8
Asthma	Widowed/Divorced	0.69 (0.6, 0.79)**	0.64 (0.53, 0.77)**	0.77 (0.63, 0.95)	0.83
Chronic pulmonary disease	Worked in the past 12 m	1.6 (1.35, 1.9) **	1.46 (1.03, 2.07)	1.6 (1.31, 1.95) **	0.91
Myocardial infarction	Household size of 3+	1.41 (1.21, 1.64) **	1.51 (1.24, 1.84) **	1.17 (0.92, 1.5)	1.29
Myocardial infarction	Worked in the past 12 m	1.39 (1.24, 1.56) **	1.19 (0.99, 1.42)	1.56 (1.34, 1.82) **	0.76 *
Stroke	Household size of 3+	1.16 (1.07, 1.27) **	1.22 (1.1, 1.35) *	1.08 (0.93, 1.27)	1.12
Arthrosis	Post-secondary education	1.17 (1.07, 1.27) **	1.35 (1.19, 1.53) **	1.02 (0.91, 1.15)	1.32 **
Arthrosis	Household size of 3+	1.46 (1.31, 1.63) **	1.42 (1.23, 1.65) **	1.47 (1.24, 1.75) **	0.96
Arthrosis	Married	0.74 (0.66, 0.83) **	0.76 (0.64, 0.9)	0.72 (0.61, 0.86) *	1.05
Arthrosis	Widowed/Divorced	0.68 (0.61, 0.77) **	0.78 (0.66, 0.93)	0.63 (0.53, 0.74) **	1.24
Arthrosis	Worked in the past 12 m	1.33 (1.23, 1.43) **	1.22 (1.09, 1.37)	1.4 (1.26, 1.55) **	0.88
Renal disease	Widowed/Divorced	0.65 (0.56, 0.76) **	0.8 (0.65, 1)	0.52 (0.41, 0.66) **	1.54 **
Depression	Household size of 3+	1.57 (1.42, 1.73) **	1.63 (1.44, 1.83) **	1.51 (1.27, 1.8) **	1.08
Depression	Married	0.75 (0.68, 0.83) **	0.77 (0.68, 0.88) *	0.72 (0.6, 0.85) *	1.08
Depression	Widowed/Divorced	0.73 (0.66, 0.8) **	0.77 (0.67, 0.87) **	0.73 (0.62, 0.85) **	1.06
GII 1					
Chronic pulmonary disease	Widowed/Divorced	0.32 (0.18, 0.58) **	0.58 (0.31, 1.1) *	0.26 (0.13, 0.53) *	2.25
Myocardial infarction	Worked in the past 12 m	1.64 (1.28, 2.09) **	1.56 (1.19, 2.03) *	2.07 (1.51, 2.85) **	0.75
Arthrosis	Widowed/Divorced	0.57 (0.46, 0.71) **	0.73 (0.55, 0.97) *	0.6 (0.45, 0.8) *	1.23
Urinary incontinence	Widowed/Divorced	0.77 (0.62, 0.97) *	0.44 (0.29, 0.67) *	0.84 (0.56, 1.27)	0.52 *
GII 2					
Arthrosis	Household size of 3+	1.31 (1.09, 1.58) **	2.09 (1.6, 2.74) **	1.19 (0.88, 1.59)	1.77 **
Arthrosis	Widowed/Divorced	0.8 (0.66, 0.98) *	0.59 (0.46, 0.76) **	0.72 (0.54, 0.95)	0.82
Arthrosis	Worked in the past 12 m	1.34 (1.18, 1.53) **	1.23 (1.05, 1.46)	1.51 (1.26, 1.8) **	0.82
Renal disease	Widowed/Divorced	0.65 (0.5, 0.84) **	0.45 (0.31, 0.65) **	0.55 (0.37, 0.82)	0.81
Depression	Widowed/Divorced	0.78 (0.66, 0.92) **	0.66 (0.53, 0.83) *	0.7 (0.53, 0.93)	0.94
GII 3					
Chronic pulmonary disease	Income Level: 3rd third	1.35 (1.07, 1.7) *	2 (1.42, 2.81) **	1.52 (1.13, 2.02)	1.32
Chronic pulmonary disease	Worked in the past 12 m	1.93 (1.46, 2.57) **	1.88 (1.3, 2.72)	1.85 (1.32, 2.6) *	1.01
Myocardial infarction	Worked in the past 12 m	1.51 (1.27, 1.78) **	1.77 (1.3, 2.39) *	1.48 (1.18, 1.86)	1.19
Arthrosis	Household size of 3+	1.55 (1.29, 1.87) **	1.15 (0.8, 1.65)	1.96 (1.45, 2.65) **	0.59 *
Arthrosis	Widowed/Divorced	0.67 (0.54, 0.83) **	0.57 (0.4, 0.81)	0.58 (0.43, 0.78) *	0.98
Arthrosis	Worked in the past 12 m	1.49 (1.3, 1.71) **	1.83 (1.47, 2.26) **	1.64 (1.36, 1.97) **	1.11
Depression	Household size of 3+	1.85 (1.54, 2.22) **	1.71 (1.17, 2.49)	1.99 (1.43, 2.77) **	0.86
Depression	Widowed/Divorced	0.69 (0.57, 0.83) **	0.8 (0.58, 1.12)	0.57 (0.42, 0.76) *	1.41
*p < 0.05, **p < 0.001					

* *p* < 0.05, ** *p* < 0.001.

## Data Availability

The data can be received from Eurostat after submitting an application.

## Supplementary Materials

The following supporting information can be downloaded at: https://www.mdpi.com/article/10.3390/jpm14080890/s1, Table S1: Survey Information.
