# Supplementary material for: Socioeconomic Gender Variables Impact the Association between Hypertension and Chronic Health Issues: Cross-Sectional Study
Source: J Pers Med. 2024 Aug 22;14(8):890. doi: 10.3390/jpm14080890 (PMC11355497; doi:10.3390/jpm14080890)
Supplement: Supplementary file 1 [file jpm-14-00890-s001.zip › jpm-3099305-supplementary.pdf]

Supplemental Material

Table S1. Survey Information.

Variable name	EHIS question
Chronic pulmonary disease	Suffering from chronic bronchitis, chronic obstructive pulmonary disease or emphysema in the past 12 months
Myocardial infarction	Suffering from a myocardial infarction (heart attack) in the past 12 months
Cardiovascular disease	Suffering from a coronary heart disease or angina pectoris in the past 12 months
Stroke	Suffering from a stroke (cerebral haemorrhage, cerebral thrombosis) in the past 12 months
Arthrosis	Suffering from arthrosis (arthritis excluded) in the past 12 months
Diabetes mellitus	Suffering from diabetes in the past 12 months
Urinary incontinence	Suffering from urinary incontinence, problems in controlling the bladder in the past 12 months
Renal disease	Suffering from kidney problems in the past 12 months
Depression	Suffering from depression in the past 12 months
